# Deep Learning Assisted Localization of Polycystic Kidney on Contrast-Enhanced CT Images

**DOI:** 10.3390/diagnostics10121113

**Published:** 2020-12-21

**Authors:** Djeane Debora Onthoni, Ting-Wen Sheng, Prasan Kumar Sahoo, Li-Jen Wang, Pushpanjali Gupta

**Affiliations:** 1 Department of Computer Science and Information Engineering, Chang Gung University, Guishan 33302, Taiwan; d0421008@cgu.edu.tw (D.D.O.); d0521006@cgu.edu.tw (P.G.); 2 Department of Medical Imaging and Radiological Sciences, Chang Gung University, Guishan 33302, Taiwan; steven.sheng@gmail.com; 3 Department of Medical Imaging and Intervention, New Taipei Municipal TuCheng Hospital, Chang Gung Medical Foundation, New Taipei City 236017, Taiwan; 4 Division of Colon and Rectal Surgery, Chang Gung Memorial Hospital, Linkou 33305, Taiwan

**Keywords:** autosomal dominant polycystic kidney disease, contrast-enhanced computed tomography, deep learning

## Abstract

Total Kidney Volume (TKV) is essential for analyzing the progressive loss of renal function in Autosomal Dominant Polycystic Kidney Disease (ADPKD). Conventionally, to measure TKV from medical images, a radiologist needs to localize and segment the kidneys by defining and delineating the kidney’s boundary slice by slice. However, kidney localization is a time-consuming and challenging task considering the unstructured medical images from big data such as Contrast-enhanced Computed Tomography (CCT). This study aimed to design an automatic localization model of ADPKD using Artificial Intelligence. A robust detection model using CCT images, image preprocessing, and Single Shot Detector (SSD) Inception V2 Deep Learning (DL) model is designed here. The model is trained and evaluated with 110 CCT images that comprise 10,078 slices. The experimental results showed that our derived detection model outperformed other DL detectors in terms of Average Precision (AP) and mean Average Precision (mAP). We achieved mAP = 94% for image-wise testing and mAP = 82% for subject-wise testing, when threshold on Intersection over Union (IoU) = 0.5. This study proves that our derived automatic detection model can assist radiologist in locating and classifying the ADPKD kidneys precisely and rapidly in order to improve the segmentation task and TKV calculation.

## 1. Introduction

Autosomal Dominant Polycystic Kidney Disease (ADPKD) is the most common hereditary renal disease with an estimated prevalence of 1:1000 to 1:2500 [1,2]. The ADPKD kidneys are characterized by continuous proliferation and growth of the bilateral renal cysts, which lead to the compression and damage of healthy renal tissue, progressive renal enlargement, and eventually progress to end-stage renal disease in majority of patients [3]. Two important biomarkers need to be examined for predicting the progressive loss of renal function: Glomerular Filtration Rate (GFR) and Total Kidney Volume (TKV) [4]. TKV allows stratification of patients into low and high-risk subgroups to identify individuals who may benefit from the treatment [3,5]. TKV is calculated using common medical imaging tests such as Computed Tomography (CT) and Magnetic Resonance Imaging (MRI). However, MRI technique is expensive and takes a long examination time of 30–60 min. Known for its faster and low-cost technique, CT has been used globally and is a popular technique in clinical imaging tests. CT can be performed with or without intravenous contrast enhancement. Contrast-enhanced Computed Tomography (CCT) highlights the blood vessels and enhances organs which provides better contrast resolution on images as compared to Non-contrast-enhanced Computed Tomography (NCCT).

**Figure 1:**
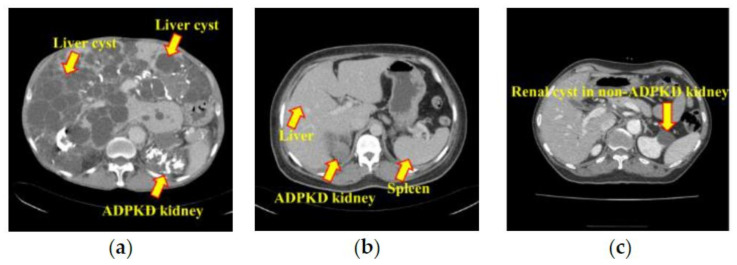
Kidney with different types of cysts: (**a**) Autosomal Dominant Polycystic Kidney Disease (ADPKD) kidney and liver cyst; (**b**) ADPKD kidney, liver, and spleen; (**c**) Renal cyst in non-ADPKD.

Precise TKV calculation requires manual localization and segmentation by clinical experts or trained personnel. Conventionally, the radiologist delineates the boundary of kidneys on images using semi-automatic tools at the medical image post-processing workstation. The use of these tools on CCT images remains a challenge with ADPKD kidney due to the contrast difference between the kidney and surrounding structure. In CT images, different body components such as air, fat, fluid, soft tissue, hemorrhage, calcification, and bones have different CT Hounsfield Units (HU) values which are displayed from low to high density. The normal kidney has homogeneous density and surrounding by the perirenal fatty tissue which shows lower density compared to soft tissue. However, in ADPKD, the kidney is replaced by multiple cysts of various densities (some of the cysts have high density due to hemorrhage or calcification), which cause the density of the kidney to become heterogeneous due to soft tissue, fluid, hemorrhage, and calcification as shown in Figure 1a. In addition, the kidney is enlarged and closely abutted to the adjacent soft tissue structure resulting in no contrast difference between the kidneys and surrounding structure. As often seen in ADPKD patients, the presence of multiple liver cysts would make segmentation of kidneys more difficult as shown in Figure 1a. It is also observed that the density of ADPKD kidney can be the same as other adjacent organs such as liver and spleen, as shown in Figure 1b. As compared to the renal cyst in non-ADPKD kidney, morphology and density of ADPKD kidney are non-uniform as shown in Figure 1c. Due to these reasons, if we want to calculate the TKV by planimetry, which is the gold standard method, the semi-automatic tool is not useful. Besides, manual segmentation of ADPKD kidney slice by slice is also a very time-consuming job.

In recent studies, computerized techniques have been widely explored for solving the challenges in medical imaging big data analysis using Artificial Intelligence (AI). Powerful AI techniques including Machine Learning (ML) [6] and Deep Learning (DL) have been used to solve problems. DL has been used in several medical applications include feature extraction, classification [7], detection [8], and segmentation [9]. One crucial application in DL is the detection or localization task. This task is essential for localizing the particular Region of Interest (ROI), which could be any nodule, cyst, tumor, or cancer in an image. It is also found that detection can be used for improving the segmentation task of ADPKD kidneys [10,11,12,13]. Therefore, the detection task can be considered as an important intermediate technique for segmentation. The detection task can be carried out in three different approaches, which could be (1) Detection by classifying a single ROI, (2) detection by locating and classifying a single ROI and (3) detection by locating and classifying multiple ROIs. One existing work [14] has used the detection by classification as an intermediate technique to detect the presence of ADPKD kidney on MRI using Convolutional Neural Network (CNN). Similarly, an ADPKD detection model has been designed using CCT images and CNN architecture which was developed based on two training sets: positive and negative kidney training patches [11]. However, the classified results only comprise information about the presence of ROI in an image without indicating the location of the ROI. In the second approach, detection is performed by classifying and locating a single ROI. However, this approach is not efficient when applied to ADPKD as we need to differentiate multiple ROIs that comprise left and right kidneys.

The object detection approach is the combination of classification and localization for detecting multiple ROIs, where it has been used as an intermediate technique for improving the segmentation task. For instance, Region with Convolutional Neural Networks (R-CNN) has been used as an object detection architecture for detecting ADPKD kidneys on MRI images with higher numbers of False Positives (FP) [15]. Several CNN architectures have been used for segmentation and object detection tasks applying Visual Geometry Group 16 layers (VGG-16), and R-CNN, respectively, on MRI [16]. Various applications have been designed using the object detection approach for different purposes. Those applications are universal lesion detection using CT images, VGG-16, and Region Proposal Network (RPN) [17], and breast cancer detection using histopathology images and Fully Convolutional Network (FCN) [18]. Furthermore, we found that less work has been done to solve the detection problem in ADPKD kidneys using an object detection approach.

Current state-of-the-art results for automated object detection can be achieved by applying several CNN DL architectures on various medical imaging techniques such as CT, CCT, NCCT, MRI, histopathology, Fundus image, colonoscopy, etc. However, the applicability of the object detection approach for localizing and classifying ADPKD kidneys using CCT images has not been thoroughly investigated. Therefore, in this work, we propose an automatic detection of ADPKD using DL on CCT images.

## 2. Materials and Methods

### 2.1. Data Acquisition

This study was approved by the Chang Gung Memorial Hospital Institutional Review Board, project identification code 201701583B0C501, approved on 18 December 2017. For this study, we collected CCT images of ADPKD patients from the Picture Archiving and Communication System (PACS) of Linkou Chang Gung Memorial Hospital from 2003 to 2019. There was a total of 110 CCT acquisitions retrieved from 97 ADPKD patients, composed of 10,078 CCT raw images. We collected one CT scan from each of 85 patients, two different CT scans from each of 11 patients, and three different CT scans from one patient. Table 1 shows the summarization of collected ADPKD patients’ gender, age, and TKV. The section thickness and interval of the collected CCT images were 5 mm and 5 mm, respectively. CCT images in Digital Imaging and Communications in Medicine (DICOM) format were displayed with a window level and width of 35 HU and 350 HU, respectively. Besides, some of the ADPKD patients with liver cysts were verified by expert radiologists.

### 2.2. Ground Truth Annotation

**Figure 2:**
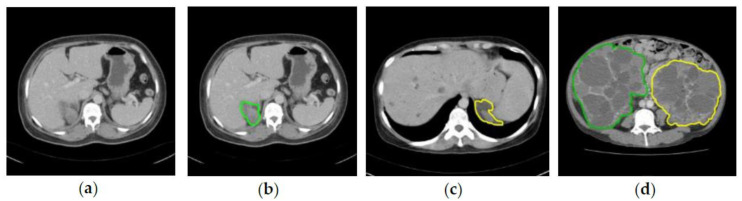
CCT raw image and respective ground truth images: (**a**) Raw image; (**b**) Ground truth for right kidney (Green); (**c**) Ground truth for left kidney (Yellow); (**d**) Ground truth for both right (Green) and left (Yellow) kidneys.

Two radiologists with more than 10 years of experience annotated the left and right kidney in CCT raw images as ground truth or gold standard. The annotation was done manually by drawing the boundary of kidneys using OSIRIX MD v10.0.5. The annotation was applied to both kidneys in all 110 CCT images. Figure 2 shows the example of CCT raw image (Figure 2a) and the respective ground truth image of right kidney (Figure 2b), left kidney (Figure 2c), and both kidneys (Figure 2d).

### 2.3. Methods

**Figure 3:**
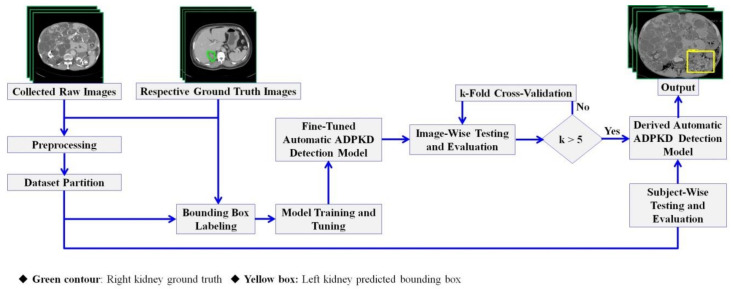
Proposed automatic ADPKD detection model framework.

In this section, we describe the proposed method, which is composed of adopted image preprocessing techniques and a Single Shot Detector (SSD) Inception V2 DL model. Figure 3 shows the proposed automatic ADPKD detection model framework.

#### 2.3.1. Preprocessing

**Figure 4:**
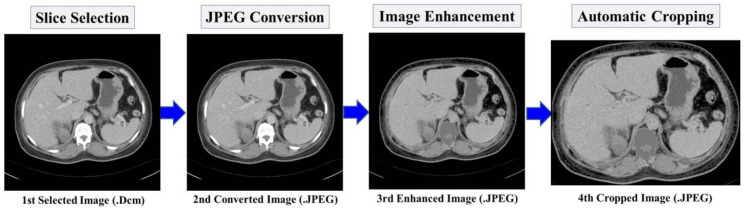
Preprocessing procedures.

The preprocessing procedure is composed of four stages, namely; (1) Slice Selection, (2) Joint Photographic Experts Group (JPEG) Conversion, (3) Image Enhancement, and (4) Automatic Cropping. We aimed to prepare the training set with less noise and eliminate the unwanted area. Figure 4 shows an example of preprocessing procedures. Firstly, some image slices were selected from the collected raw images in DICOM format based on the inclusion and exclusion criteria. The selection criterion was decided based on the presence of either left or right kidney in the images. Thus, total number of collected raw images was reduced from 10.078 to 4648 images. Secondly, all selected images were converted to JPEG format using open source software such as RadiAnt DICOM Viewer without changing the dimension, quality of raw images, and disabling patient’s information. Thirdly, the image enhancement method was applied to reduce the noises such as high-density organs in the spine, ilium, and cysts or kidney stones. To cope with variant of intensity, an intensity segmentation based approach was chosen as in [19], where a global thresholding method was adopted. Based on our experiment and observation, we found the preferable value of threshold T_max_ = 195 and T_min_ = 45. The thresholding operation can be expressed as follows:

(1) $$\mathrm{dst}\left(x,y\right)=\left\{\begin{array}{c} \begin{array}{cc} \mathrm{MaxVal} & \mathrm{if}\;\mathrm{src}\left(x,y\right)>T_{\max}\\ 0 & \;\mathrm{if}\;\mathrm{src}\left(x,y\right)<T_{\min} \end{array} \end{array}\right\},$$

where, MaxVal = 120 denotes maximum pixel intensity, src(x, y) in the source image pixel intensity, and dst(x, y) refers to the destination image pixel intensity. Fourthly, an automatic cropping mechanism was designed to avoid bias by eliminating unimportant parts in the image without modifying the dimension. As shown in Figure 4, kidneys are located in center of the abdominal cavity surrounded by the black pixel intensity, where src(x, y) = 0. Dilation morphological [20] method was applied to emerge the shape of abdominal cavity. Considering the enhanced image as a binary input image B, and kernel size S = 4 × 4, the dilation of B ⊕ S was performed. Moreover, we automatically cropped the whole abdomen cavity area by finding the maximum contour area [21] using hierarchy contour. Then rectangular shape was drawn by taking the maximum area of the contour. Lastly, we cropped the rectangular area by using the coordinates ymin(y): ymax(y + h), xmin(x):xmax(x + w), where x, y are coordinates, and h, w represents the height and width, respectively.

#### 2.3.2. Dataset Partition

Initially, the dataset of 110 subjects (e.g., 4648 slices) is partitioned randomly into training and testing set with a ratio of 80:20. Accordingly, 88 subjects (i.e., 3718 slices) and 22 subjects (i.e., 930 slices) were selected for the training and testing set, respectively. Then, we carried out the model training, tuning, and testing. It is to be noted that the set of images in the training set were not included in the testing set. The 88 subjects (i.e., 3718 slices) of the training set were used to train and tune the model. We performed the image-wise testing and evaluation using 5-fold cross validation [22], where each k_i_, an element of k-subsets, will have an approximately equally sized image, resampled randomly from all considered subjects. Then, we assigned randomly each k_i_ for the testing set and the rest (k1) were assigned for the training set. For all k-rounds of training and testing, the final image-wise testing results were obtained from the average results of k_i_ testing sets. This technique is used to assess the overfitting problem and evaluate the robustness of our fine-tuned trained model, as given in Section 2.3.5. Lastly, to test our derived detection model with the unseen data, we performed subject-wise testing on 22 subjects (i.e., 930 slices) testing set.

#### 2.3.3. Bounding Box Labeling

Bounding box labeling was performed after completing the preprocessing procedure as given in Section 2.3.1 and partitioning the dataset as described in Section 2.3.2. this is required to learn the coordinates and classes of the kidneys during the training process. The respective ground truth images were used as reference for the bounding box labeling. The labeling was carried out by drawing boxes around the kidneys and assigning classes within an image using open source LabelImg software v1.8.4 [23]. By doing so, the coordinates and classes of the kidneys were saved in Extensible Markup Language (XML) files in PASCAL VOC format. To verify our bounding box labeled with the annotated ground truth, we redrew the bounding box on the annotated ground truth. Then, we checked the coordinates, where our derived bounding box labeled coordinates should be greater than the annotated ground truth coordinates.

#### 2.3.4. Automatic ADPKD Detection Model

**Figure 5:**
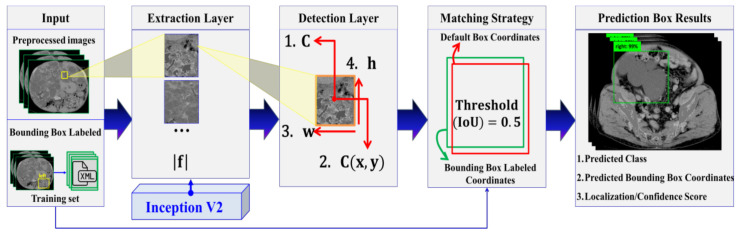
The architecture of automatic ADPKD detection model, where $\left|f\right|$, C(x, y), w, h, and V2 refer to as total number of feature maps, center bounding box, width, height, and version 2, respectively.

We proposed an automatic ADPKD detection model using a DL object detection approach, specifically a regression-based approach. We selected SSD [24] Inception V2 [25] as the adopted network based on pre-trained model performance speed, using the Microsoft Common Objects in Context (COCO) dataset [26], a large-scale dataset for DL applications such as object detection, segmentation, and captioning. Based on the single feed-network approach, SSD comprises two main layers: extraction layer and detection layer. SSD is categorized as a one stage detector, where the detection is performed directly after extracting the feature maps through the CNN backbone. Thus, SSD can work faster as compared to a three stages detector such as Viola and Jones, Histogram of Oriented Gradient (HOG), etc., and a two stages detector such as R-CNN, Faster R-CNN, etc. The architecture of the proposed detection model is illustrated in Figure 5.

In input layer, all preprocessed images of size = 224 × 224 and the corresponding labeled bounding box coordinates of XML files were fed into the extraction layer, where the total number of feature maps |f| was extracted using a pre-trained Inception V2 CNN network. The feature maps were extracted by implementing minimum depth of convolutional layers = 16 and the Rectified Linear Unit (ReLU) activation function. In the next step, each extracted feature map was passed to the detection layer, where four categories need to be detected: the total number of classes C, center bounding box C(x, y) coordinates, width w, and height h. To detect these four categories, default detector boxes were used in each feature map, where different scales, scale max = 0.2, scale min = 0.95, and a set of aspect ratio = {1, 2, 3, 1/2, 1/3} were considered in our implementation. The detection was implemented with total number of default boxes = 4 and kernel size 3 × 3. Furthermore, a matching strategy was applied based on the best fit in terms of aspect ratio, scale, and location. The comparison was based on the Jaccard index or Intersection over Union (IoU), where threshold = 0.5 was considered in our experiment. For a particular class, a matching method was carried out by comparing the labeled bounding box with all generated default boxes’ coordinates as shown in Equation (2).

(2) $$\begin{array}{c} \mathrm{Match}(\mathrm{Bounding}\;\mathrm{Box}\;\mathrm{Labeled}\left[\mathrm{Ymin}\left(y\right):\mathrm{Ymax}\left(y+h\right),\mathrm{Xmin}\left(x\right):\mathrm{Xmax}\left(x+w\right)\right],\\ \mathrm{Default}\;\mathrm{Boxes}\left[\mathrm{Ymin}\left(y\right):\mathrm{Ymax}\left(y+h\right),\mathrm{Xmin}\left(x\right):\mathrm{Xmax}\left(x+w\right)\right]), \end{array}$$

Based on the comparison, prediction boxes were produced, where each predicted box comprises the predicted class, predicted bounding box coordinates [Ymin(y):Ymax(y + h), Xmin(x):Xmax(x + w)] and localization or confidence score. The prediction results were categorized into three metrics: True Positive (TP) if localization score ≥ threshold with correct classification, False Positive (FP) if localization score < threshold with wrong classification, and False Negative (FN) if labeled bounding box is not detected by the model. Moreover, Non-Maximum Suppression (NMS) was applied to find the best prediction box results for each class. The smooth L1 was used for localization loss L_lo_ and Softmax was applied for classification loss L_co_.

#### 2.3.5. Training and Tuning Model

We performed the training and tuning model without cross-validation strategy on 88 subjects (i.e., 3718 slices) of training data. As reported in [27], cross-validation is known as internal validation. Using both techniques together can lead to high variance and non-optimized hyper-parameters. Moreover, the random search technique, one of the hyper-parameter tuning strategies which outperforms the grid search technique, was applied. To improve the robustness of our designed model, selection of the best hyper-parameters is performed through model training. Based on our experiment, a fine-tuned trained model was achieved by setting the optimal value of the hyper-parameters: initial learning rate = 0.004, decay = 0.9, epsilon = 0.001, momentum optimizer = 0.9, batch size = 24, and step = 6000. Furthermore, two data augmentation techniques, random horizontal flip, where an input image is flipped from left to right with probability of 0.5, and SSD random crop, where subsets of patches are cropped from an input image randomly, were applied.

#### 2.3.6. Image-Wise and Subject-Wise Testing and Evaluation

To verify how robust our fine-tuned trained model is, image-wise testing and evaluation using 5-fold cross-validation were performed, with k-rounds of training and testing, where one fold $k_{i}$ was assigned to the testing set in each round, and the remaining (k − 1) subsets were assigned to the training sets. After testing and evaluating our fine-tuned trained model, performance of detection was evaluated by taking the average evaluation metrics of whole k-rounds. Lastly, we assessed and evaluated subject-wise testing to finally test our fine-tuned trained model using 22 subjects (i.e., 930 slices).

### 2.4. Experimental Setup

The automatic ADPKD detection model was built using the OpenCV v3.2.0 image preprocessing tool for image enhancement and automatic cropping, and TensorFlow-GPU v1.12 image analysis tool for detection model. Python v3.6.9 programming language was used as an interface for both tools. Moreover, high-performance hardware and software are required to execute the experiments. The hardware specification was GPU TITAN RTX 24 GB × 4 and 256 GB memory. For software, we used Ubuntu v18.04.3 operating system including python libraries Pandas v1.0.5, Numpy v1.19.1, Matplotlib v3.3.0, Pillow v7.2.0, osmnx v0.15.1, lxml v4.2.1, imageio v2.5.0, Urllib3 v1.22, Sys v3.6.9, and Zipfile v0.5.1.

### 2.5. Evaluation Metrics

IoU was used to evaluate the performance of our ADPKD detection model. This metric is commonly used for object detection and segmentation evaluation, where IoU is defined by the intersection between predicted box coordinates and labeled bounding box coordinates divided by their union. Based on IoU calculation, TP, FP, and FN can be defined. Moreover, metrics including Accuracy, Localization loss (L_lo_), Classification loss (L_co_), Precision, Recall/Sensitivity, and F1-Score were considered and calculated as follows:

(3) $$\mathrm{Accuracy}=\frac{\mathrm{TP}}{\mathrm{TP}+\mathrm{FP}+\mathrm{FN}}$$

(4) $$\mathrm{Precision}=\frac{\mathrm{TP}}{\mathrm{TP}+\mathrm{FP}}$$

(5) $$\mathrm{Recall}/\mathrm{Sensitivity}=\frac{\mathrm{TP}}{\mathrm{TP}+\mathrm{FN}}$$

(6) $$F1-\mathrm{Score}/\mathrm{Dice}-\mathrm{Score}=2\times \frac{\left(\mathrm{Precision}\times \mathrm{Recall}\right)}{\left(\mathrm{Precision}+\mathrm{Recall}\;\right)}$$

After defining other metrics, Average Precision (AP) and mean AP (mAP) were calculated. Both metrics were calculated based on precision-recall calculation metrics. The precision-recall curve can be plotted graphically into a 2D graph, where X-axis denotes the recall and Y-axis represents the precision range [0, 1]. AP for predicted class can be calculated by finding precision-recall Area Under the Curve (AUC). Based on the AP value, mAP value can be calculated by taking average AP of both kidney classes. In each round, precision and recall were calculated as given in Equations (4) and (5) with IoU threshold set to be 0.5. Then AP and mAP values were computed. These procedures were carried out until k-rounds were completed.

### 2.6. Evaluation Procedures

Firstly, we performed image-wise testing and evaluation on our fine-tuned ADPKD detection model using k-fold cross-validation technique. Secondly, we conducted subject-wise testing. Both testing evaluations were carried out by comparing our method with other well-established DL pre-trained object detection models including CNN backbones, which have been adopted in similar current approaches for pneumonia detection in chest X-ray [28] and for malignant pulmonary nodule detection in CT scans [29]. These models and backbones were SSD Inception V2, Faster-RCNN, ResNet, and MobiNet. Alongside, we included DL object detection models, which have been trained using large-scale datasets including COCO, Kitti, Open Image, etc., developed by TensorFlow [30]. Correspondingly, we selected models such as original SSD Inception V2, SSD MobileNet V1, Faster R-CNN NAS, Faster R-CNN Inception ResNet V2, and R-FCN ResNet 101. We trained all selected pre-trained models by partitioning the data set as given in Section 2.3.2 and evaluated using k-fold cross validation techniques as given in Section 2.3.6.

## 3. Results

### 3.1. Evaluation Results of Image-Wise Testing

We conducted the experiments on image-wise testing, and compared the results with other detection architectures as shown in Table 2. It was observed that our model achieved the highest performance in various evaluation metrics with an accuracy of 0.90 for right and 0.91 for left kidney. It was also found that our model outperformed over other architectures with an optimal confidence F1-Score of 0.86 for right kidney and 0.88 for left kidney.

**Figure 6:**
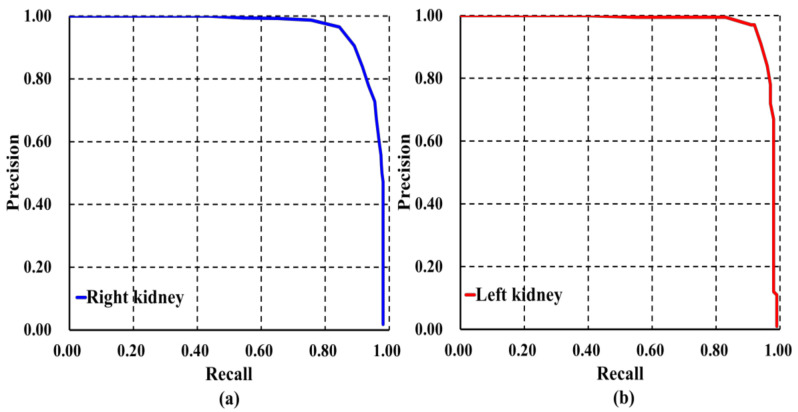
Precision-recall curve of our model on image-wise testing set: (**a**) Right kidney; (**b**) Left kidney.

In addition to different evaluation metrics, AP and mAP were calculated to analyze the robustness of our localization model, the results of which are presented in Table 3 and Figure 6. It is observed that our model was able to locate and classify both kidneys with AP right = 0.934 and AP left = 0.944 as shown in Figure 6a,b, respectively. We also found that our model was able to localize and classify both kidneys with the highest value of mAP = 0.94 in comparison to other well-established detection architectures.

**Figure 7:**
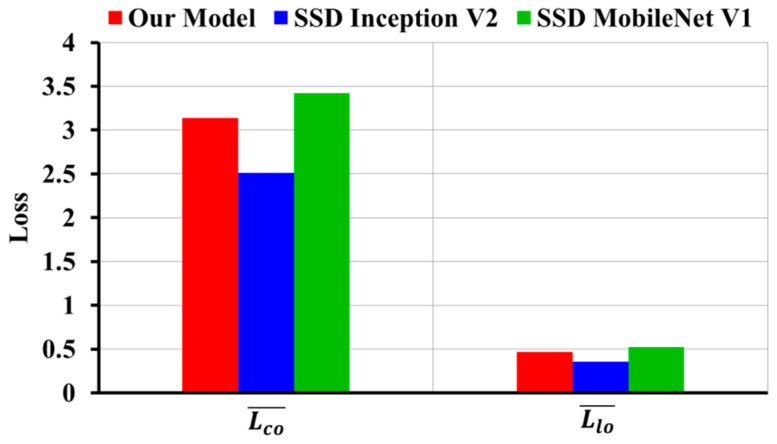
Comparison of classification and localization loss on image-wise testing set.

It is observed that our model has higher average value of Classification loss $\bar{L_{\mathrm{co}}}$ = 3.137 and Localization loss $\bar{L_{\mathrm{lo}}}$ = 0.464 as compared to the original SSD Inception V2 Classification loss $\bar{L_{\mathrm{co}}}$ = 2.511 and Localization loss $\bar{L_{\mathrm{lo}}}$ = 0.353. However, our model has lower average value of Classification and Localization loss as compared to SSD MobileNet V1, where SSD MobileNet V1 Classification loss $\bar{L_{\mathrm{co}}}$ = 3.423 and Localization loss $\bar{L_{\mathrm{lo}}}$ = 0.52. Figure 7 shows the comparison of loss calculation graphically.

**Figure 8:**
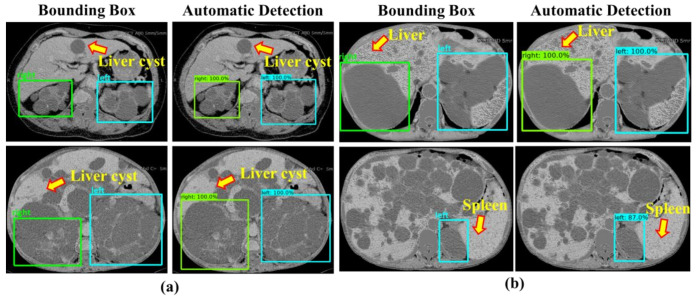
Detection results: (**a**) ADPKD kidneys associated with liver cysts; (**b**) ADPKD kidneys with adjacent organs.

Figure 8a shows the example of results generated by our derived ADPKD detection model on CCT. It can be seen that our model works robustly though ADPKD kidneys are heterogeneous in density, in particular when multiple liver cysts are present. Similarly, adjacent to the liver and spleen, our model was able to localize and classify the kidneys as shown in Figure 8b.

### 3.2. Evaluation Results of Subject-Wise Testing

Upon performing the image-wise testing, we conducted subject-wise testing. As reported in Table 4, our proposed model outperformed all object detection architectures, where the average of all metrics is 0.8 for right kidney and 0.816 for left kidney.

**Figure 9:**
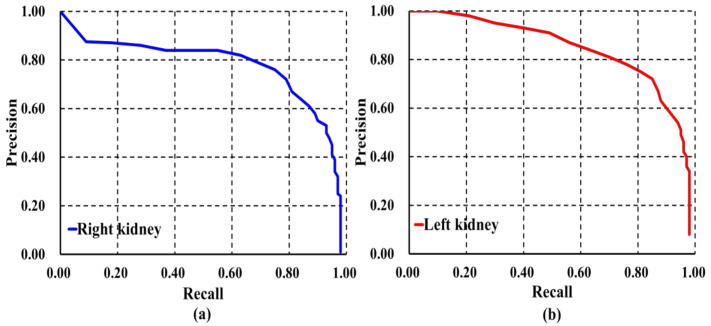
Precision-recall curve of our model on subject-wise testing: (**a**) Right kidney; (**b**) Left kidney.

These results were supported by other values of the evaluation metrics as shown in Table 5. It is evidently observed that our model can detect and classify ADPKD kidneys with an AP of 0.80 for right kidney and 0.852 for left kidney, shown in Figure 9a,b, respectively. As compared to other detection architectures, our model achieved the highest mAP, 0.824.

**Figure 10:**
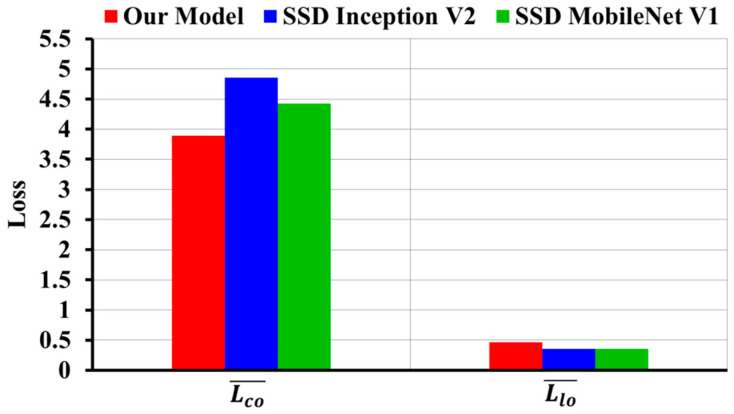
Classification and localization loss comparison on subject-wise testing set.

In addition to the loss calculation, we analyzed and plotted the Classification loss and Localization loss as shown in Figure 10. It was observed that our model has lower average value of Classification loss $\bar{L_{\mathrm{co}}}$ = 3.89 as compared to SSD Inception V2 $\bar{L_{\mathrm{co}}}$ = 4.859 and SSD MobileNet V1 $\bar{L_{\mathrm{co}}}$ = 4.424, though Localization loss of our model was $\bar{L_{\mathrm{lo}}}$ = 0.463.

**Figure 11:**
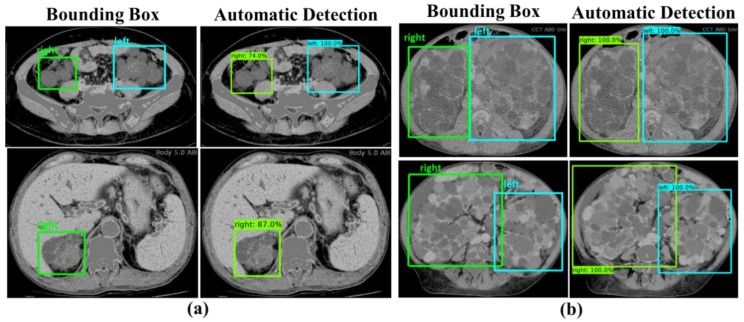
Detection results: (**a**) Small size of ADPKD kidneys; (**b**) Big size of ADPKD kidneys.

Furthermore, the localized outputs obtained from the subject-wise testing are depicted in Figure 11a,b. It was observed that our model could detect and classify either a small or big size of the ADPKD kidneys.

## 4. Discussion

In this paper, we demonstrated that the derived automatic ADPKD detection model of CCT images is robust. We performed image-wise testing evaluation on our fine-tuned trained detection model using a k-fold cross-validation technique and compared it with other pre-trained models. It is found that our fined-tuned model outperformed other pre-trained object detection models, though pre-trained models have been designed using large-scale images [31]. Our model can localize and classify ADPKD kidneys with mAP = 0.94. To verify the robustness of our derived detection model, we conducted subject-wise testing evaluation, where our model can obtain mAP = 0.824. It was observed that our model works precisely, using CCT images instead of using MRI images for detecting ADPKD kidneys [15]. Although only 370 subjects for training and 78 for testing have been considered in this work [11], our model has several advantages in solving ADPKD kidney detection without segmentation. These advantages can be described from clinical and technical points of view. From the clinical point of view, our detection model can automatically locate and classify kidneys associated with multiple liver cysts though it has been found that automatic ADPKD kidney segmentation using CCT has over-estimated in the presence of liver cysts [10]. In addition, the automatic detection model can localize the kidney’s area, which overlaps with adjacent organs such as liver and spleen. Therefore, it was also proved from the results that our detection model can work robustly in the case of non-uniform morphology and density of ADPKD kidneys.

By minimizing time-consuming steps and reducing the labor of radiologists, our model achieved a fewer number of False Positives with AP = 0.939 on image-wise testing and AP = 0.826 on subject-wise testing for both classes as compared to [15], where the AP value was 0.78 for both classes. Thus, our model can perform well in assisting radiologists in locating ADPKD kidneys, calculating the TKV and reducing the time-consuming steps.

From the technical point of view, our derived detection model can successfully be implemented on CCT images. Moreover, our model can replace any manual cropping procedure or software that is used to crop the ROI of kidneys and become an intermediate technique before segmentation. Based on our experiment, we also discovered that the accuracy of our detection model could be optimized by training numerous CCT images, of varied morphology and density, of the ADPKD kidneys.

**Figure 12:**
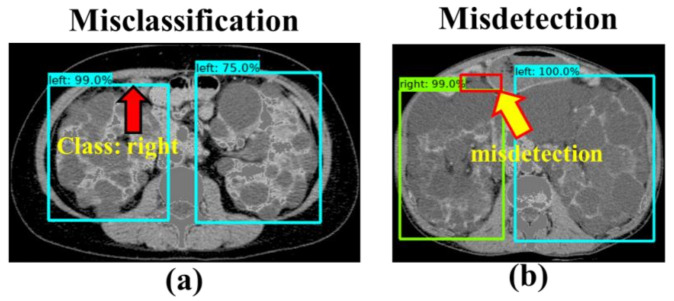
Misclassification and misdetection example: (**a**) Misclassification; (**b**) Misdetection.

However, our study has limitations. In the testing phase, a high average of losses, misclassification and misdetection can be found. Although it can classify correctly as a “right” kidney, the bounding box is not precisely located in the right kidney. Based on these characteristics, some misclassification and misdetection id inevitable as shown in Figure 12a,b. In addition to data acquisition, repeated scans have been performed to collect the CCT slices from the same subject. Therefore, we plan to conduct more experiments with greater numbers of NCCT and CCT images from ADPKD patients through independent scans. Although our model can locate and classify the ROI of ADPKD kidneys, exact kidney area cannot be extracted perfectly. It is to be noted that, though our model works precisely in detecting the bounding box that can cover all ADPKD kidney areas adjacent to the liver cysts, a few portions of the liver cysts could still be included in the predicted bounding box. Therefore, we plan to extend this work to exactly localize and distinguish the ADPKD kidneys from the liver cysts by segmenting the ADPKD kidney areas. We plan to use our automatic ADPKD detection model as an intermediate technique before segmentation to improve the accuracy of ADPKD segmentation and achieve precise calculation of TKV. For further confirmation of this proposed model, we plan to conduct the experiment with prospective data in future.

## 5. Conclusions

In this study, an automatic detection model for ADPKD kidney on CCT images is designed. The designed model is built using image preprocessing and Deep Learning techniques. We used various image preprocessing techniques such as global thresholding, morphological dilation operation, and contouring. We also adopted and tuned the DL SSD Inception V2 architecture. Based on the performance evaluation, our model outperformed other well-established detection architectures with mAP of 94% for image-wise testing, and mAP of 82% for subject-wise testing. Furthermore, our contribution is to design a robust automatic ADPKD kidneys detection model on CCT images, which can accelerate manual localization and classification tasks with higher accuracy and can establish an intermediate technique for segmentation task. Hence, we believe that the automatic detection model for ADPKD could be a promising intermediate technique in assisting the radiologists to locate, segment, and analyze ADPKD kidneys for TKV measurement on CCT image big data.

## Figures and Tables

**Table 1:** 110 Contrast-enhanced Computed Tomography (CCT) acquisitions from 97 ADPKD patients.

Characteristic		Mean ± SDs (Range) or Number
Age at examination (yrs)		54.59 ± 18.5 (27–88)
Sex	Male	52
	Female	45
TKV (cm^3^)		2734.33 ± 2312.45 (345.14–13,666.88)

**Table 2:** Evaluation metrics results on image-wise testing.

Detection Architectures	Class	Evaluation Metrics			
		Accuracy	Precision	Recall	F1-Score
Our Model	right	0.90 (±0.06)	0.92 (±0.07)	0.82 (±0.02)	0.86 (±0.04)
	left	0.91 (±0.06)	0.92 (±0.06)	0.84 (±0.06)	0.88 (±0.05)
Single Shot Detector (SSD) Inception V2	right	0.86 (±0.04)	0.90 (±0.03)	0.80 (±0.04)	0.84 (±0.02)
	left	0.86 (±0.04)	0.91 (±0.03)	0.82 (±0.03)	0.86 (±0.03)
SSD MobileNet V1	right	0.73 (±0.1)	0.75 (±0.01)	0.72 (±0.1)	0.72 (±0.09)
	left	0.71 (±0.1)	0.81 (±0.06)	0.66 (±0.1)	0.71 (±0.1)
Faster Region with Convolutional Neural Networks (R-CNN) NAS	right	0.57 (±0.1)	0.51 (±0.04)	0.69 (±0.07)	0.58 (±0.01)
	left	0.46 (±0.06)	0.69 (±0.02)	0.43 (±0.06)	0.52 (±0.04)
Faster R-CNN Inception ResNet V2	right	0.27 (±0.05)	0.43 (±0.09)	0.26 (±0.09)	0.31 (±0.07)
	left	0.52 (±0.04)	0.50 (±0.02)	0.67 (±0.1)	0.57 (±0.06)
Region-FCN (R-FCN) ResNet 101	right	0.37 (±0.09)	0.57 (±0.1)	0.34 (±0.08)	0.42 (±0.07)
	left	0.53 (±0.08)	0.58 (±0.05)	0.67 (±0.06)	0.62 (±0.05)

NAS: Neural Architecture Search.

**Table 3:** Comparison of Average Precision (AP) and mean Average Precision (mAP) with other architectures on image-wise testing.

Detection Architectures	Evaluation Metrics (Average (±SD))		
	AP		mAP
	Right	Left	Both Classes
Our Model	0.934 (±0.01)	0.944 (±0.01)	0.94 (±0.01)
SSD Inception V2	0.844 (±0.03)	0.866 (±0.04)	0.855 (±0.03)
SSD MobileNet V1	0.679 (±0.10)	0.705 (±0.10)	0.692 (±0.09)
Faster R-CNN NAS	0.594 (±0.03)	0.625 (±0.03)	0.609 (±0.03)
Faster R-CNN Inception ResNet V2	0.491 (±0.08)	0.568 (±0.06)	0.53 (±0.07)
R-FCN ResNet 101	0.506 (±0.11)	0.649 (±0.11)	0.578 (±0.11)

**Table 4:** Evaluation metrics results of subject-wise testing.

Detection Architectures	Class	Evaluation Metrics			
		Accuracy	Precision	Recall/Sensitivity	F1-Score
Our Model	right	0.8	0.8	0.8	0.8
	left	0.817	0.8	0.84	0.81
SSD Inception V2	right	0.434	0.552	0.512	0.531
	left	0.511	0.582	0.632	0.606
SSD MobileNet V1	right	0.563	0.574	0.652	0.611
	left	0.549	0.654	0.558	0.602
Faster R-CNN NAS	right	0.483	0.394	0.728	0.511
	left	0.248	0.549	0.232	0.326
Faster R-CNN Inception ResNet V2	right	0.268	0.291	0.266	0.278
	left	0.513	0.464	0.694	0.556
R-FCN ResNet 101	right	0.3	0.464	0.245	0.332
	left	0.529	0.572	0.676	0.620

**Table 5:** AP and mAP comparison with other architectures on subject-wise testing.

Detection Architectures	Evaluation Metrics		
	AP		mAP
	Right	Left	Both Classes
Our Model	0.80	0.852	0.824
SSD Inception V2	0.42	0.497	0.458
SSD MobileNet V1	0.567	0.536	0.551
Faster R-CNN NAS	0.48	0.549	0.515
Faster R-CNN Inception ResNet V2	0.483	0.612	0.548
R-FCN ResNet 101	0.474	0.681	0.577
